# Characteristic chest CT findings for progressive cavities in *Mycobacterium avium* complex pulmonary disease: a retrospective cohort study

**DOI:** 10.1186/s12931-020-1273-x

**Published:** 2020-01-08

**Authors:** Yohei Oshitani, Seigo Kitada, Ryuya Edahiro, Kazuyuki Tsujino, Hiroyuki Kagawa, Kenji Yoshimura, Keisuke Miki, Mari Miki, Hiroshi Kida

**Affiliations:** 1 Department of Respiratory Medicine, National Hospital Organization, Osaka Toneyama Medical Center, 5-1-1 Toneyama, Toyonaka-shi, Osaka, 560-8552 Japan; 2 Department of Respiratory Medicine, Yao Tokushukai General Hospital, Osaka, Japan; 3 Department of Respiratory Medicine and Clinical Immunology, Osaka University Graduate School of Medicine, Osaka, Japan

**Keywords:** Nontuberculous mycobacteria, Predictor, Cavity, *Mycobacterium avium* complex, Computed tomography

## Abstract

**Background:**

Although cavities are an important finding in *Mycobacterium avium* complex pulmonary disease (MAC-PD), there is little information regarding the types of cavities that indicate disease progression. This study was performed to identify cavity characteristics that were associated with disease progression in patients with MAC-PD.

**Methods:**

This retrospective cohort study included 97 patients presenting with MAC-PD with cavities between December 2006 and June 2016. We compared initial and final computed tomography (CT) findings, classified 52 and 45 patients in the progressive and non-progressive cavity groups, respectively, and examined the progression-related imaging features in initial CT images. A progressive cavity was defined by more than two-fold increase in internal diameter or emergence of a new cavity around the initial cavity.

**Results:**

Patients in the progressive group were older (*p* < 0.001), had a lower body mass index (*p* = 0.043), and showed higher diabetes complication rates (*p* = 0.005). The initial CT in the progressive group showed a longer maximum internal diameter of the cavity (*p* < 0.001) and higher rates of cavities close to the chest wall (*p* < 0.001), multiple cavities (*p* = 0.023), consolidation around the cavity (*p* < 0.001), atelectasis (*p* = 0.011), and pleural thickening (*p* < 0.001). Multivariable logistic regression analysis revealed that the maximum internal diameter of the cavity (odds ratio [OR]: 1.11, 95% confidence interval [CI]: 1.02–1.21; *p*=0.012) and consolidation around the cavity (OR: 16.15, 95% CI: 4.05–64.46; *p* < 0.001) were significantly associated with progressive cavities. In cavities with a maximum internal diameter of ≥10 mm and simultaneous consolidation, the probability of progression was as high as 96.2%. The 10-year mortality rates in the progressive and non-progressive cavity groups were 46.7 and 9.8% (*p* < 0.001), respectively, while the 10-year respiratory failure rates were 28.1 and 0%, respectively (*p* < 0.001).

**Conclusions:**

Large cavity size and consolidation on CT showed strong relationships with disease progression, which led to respiratory failure and high mortality rate.

## Background

The prevalence of nontuberculous mycobacteria pulmonary disease (NTM-PD) is reportedly increasing worldwide. The annual prevalence in the United States significantly increased from 20 to 47 patients/100,000 persons between 1997 and 2007 [1, 2]. Although the incidence of this disease in Europe is low, it is also steadily increasing [3, 4]. A similar trend was observed in Japan, where the annual prevalence significantly increased from 6.7 to 14.7 patients/100,000 persons between 2005 and 2014 [5]. *Mycobacterium avium* complex (MAC) is the most frequently identified pathogen in a report summarizing the frequency of NTM isolation in the world [4]. Thus, MAC pulmonary disease (MAC-PD) is the most important disease among NTM infections.

Poor prognostic factors for MAC-PD include old age, low body mass index (BMI), low lung function, anemia, high blood deposition, malignancy, and hemosputum. Notably, the presence or absence of cavities is the most important factor [6–9]. Enlarged progressive cavities destroy lungs, leading to respiratory failure and poor prognosis [10, 11]. The prognosis in fibrocavitary (FC) disease, which is characterized by cavities on the lung apex, is significantly worse than that in nodular/bronchiectatic (NB) disease, which is characterized by nodules and bronchiectasis in the middle lobe and lingula. The total 10-year mortality rate in 634 patients with MAC-PD was 74.8% in patients with cavities and 34.8% in those without cavities [6]. Cavities also appear in patients with progressive NB disease, resulting in poor prognosis. The 10-year mortality rate in 782 patients with NB MAC-PD was 25.1% in those with cavities and 0.8% in those without cavities [8].

The British Thoracic Society guidelines [12] recommend surgery in cases with cavitary disease that is limited in site and extent; therefore, control of cavitary lesions is important. However, when the cavity is progressive and the lung destructive lesion enlarges, surgical intervention becomes difficult in clinical practice. According to a long-term observation of 125 cases involving pulmonary resection for NTM-PD, pneumonectomy and remnant cavitary lesions after surgery were found to be significant predictive factors for microbiological recurrence and survival [13]. Therefore, it is important to recognize progressive cavities at an earlier stage. However, it is difficult to predict cavity progression because some cavities progress while others show a relatively stable course.

We conducted a retrospective cohort study to evaluate chest computed tomography (CT) findings and the progression of cavitary lesions in patients with MAC-PD in order to clarify the types of cavities that are likely to progress at an early stage. If these can be clarified, the findings may facilitate the identification of treatment plans.

## Methods

### Selection of study subjects

A total of 485 outpatients with MAC-PD, diagnosed on the basis of the diagnostic criteria for NTM-PD advocated by the America Thoracic Society/Infectious Disease Society of America in 2007 [14], were identified between December 2006 and June 2016 at National Hospital Organization, Osaka Toneyama Medical Center. Among these, we extracted the data of 139 patients who had cavities, were observed for over 3 years, and could be evaluated with CT at two or more points. A cavity was defined as a radiographic opacity with an internal area of lucency. Beaded airspace enlargements that were apparently contiguous with the airways were excluded as bronchiectasis. We also excluded patients who underwent lung resection, or who were associated with lung cancer, interstitial pneumonia, or pulmonary aspergillosis because these diseases would be considered the primary condition rather than MAC-PD in these cases. Thus, 97 patients were finally enrolled into this study.

The study was approved by the National Hospital Organization, Osaka Toneyama Medical Center Review Board (approval number TNH-2019005), Osaka, Japan. The approval allowed retrospective data collection and reporting of anonymous results without acquisition of informed consent from eligible study subjects.

### Definition of a progressive cavity

**Fig. 1:**
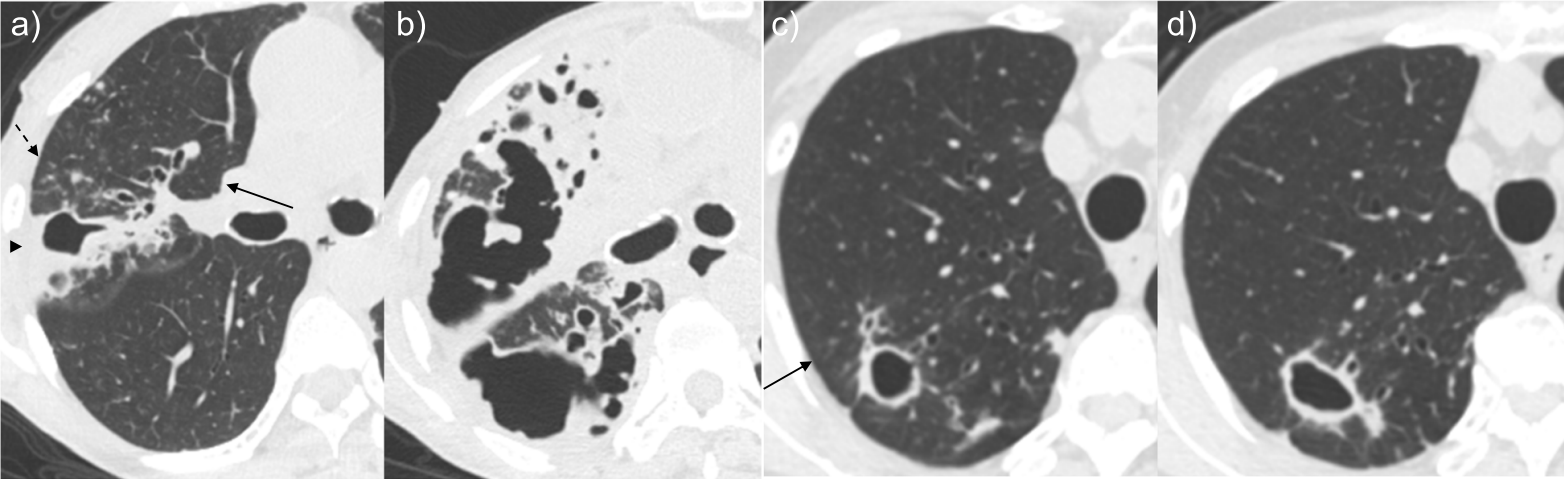
Typical computed tomography (CT) findings for *Mycobacterium avium* complex pulmonary disease patients in this study. **a**, **b** A patient in the progressive cavity group. The follow-up period was 4.2 years. **A**) Initial CT scan (section thickness, 1 mm) showed cavity formation (maximum inner diameter, 22 mm) with consolidation (arrowhead), a small nodule, a nodule (dashed arrow), and bronchiectasis (arrow) in the right upper lobe. **b** Follow-up CT scan (section thickness, 1 mm) at a similar level showed extension of the cavity and emergence of new cavities. **c**, **d** A patient in the non-progressive cavity group. The follow-up period was 3 years. **c** Initial CT scan (section thickness, 1 mm) showed cavity formation (maximum inner diameter, 14 mm) with bronchiectasis (arrow) in the right upper lobe. **d** Follow-up CT scan (section thickness, 1 mm) at a similar level showed cavity formation (maximum inner diameter, 21 mm). All images show lung tissue (window width, 1500 Hounsfield units (HU); window level, − 600 HU)

Initial CT was defined as the CT examination on which a cavity was first identified, and the corresponding cavity was defined as the initial cavity. The size of this cavity at the time of the final observation was compared to its initial size, and the patients were classified into the progressive or non-progressive cavity groups accordingly. If a patient had multiple cavities, the cavity exhibiting the largest internal diameter at the time of cavity confirmation was evaluated. A progressive cavity was defined by a more than two-fold increase in internal diameter in comparison with the initial cavity size or the emergence of a new cavity around the initial cavity (Fig. 1a, b). The non-progressive cavity group included all cases other than those in the progressive cavity group (Fig. 1c, d).

### Clinical assessment

Baseline clinical characteristics were obtained from medical chart review, including patient observation period (the interval between the initial visit and the final observation), cavity observation period (the interval between the initial and the final CT), sex, age at initial cavity confirmation, BMI, underlying lung disease, comorbidities, steroid and/or immunosuppressive agent use, MAC species, sputum-smear positivity at initial cavity confirmation, treatment, levels of glycopeptidolipid-core antibody, the rate of all-cause death, and respiratory failure as prognosis. A multidrug regimen was defined as the use of two or more drugs containing macrolide antibiotics for at least 1 year. Respiratory failure was defined as initiation of home oxygen therapy and/or noninvasive pressure ventilation therapy.

### Chest CT assessment

In the initial cavity assessment, the lung area where the cavity was present, the largest internal diameter, and the shortest distance from the pleura to the outer cavity wall were evaluated. The presence or absence of multiple cavities was also evaluated. We defined the cavity that was completely tangential to the pleura as the cavity close to the chest wall. Pulmonary areas were divided into three regions: upper lobe, middle lobe or lingula, and lower lobe. Among CT findings around the initial cavity, bronchiectasis, small nodules (≤ 5 mm), nodules (> 5 mm and < 30 mm), consolidation, atelectasis, pleural thickening, pleural indentation, pulmonary emphysema, or presence of bulla were evaluated. Chest CT findings were assessed by two pulmonologists blinded to the clinical data. Discrepancies were solved through a consensus review.

### Statistical analysis

Continuous data were summarized as quartiles and compared using the Wilcoxon rank-sum test for two-group comparisons. Categorical data were summarized as numbers (percentages) and compared using Fisher’s exact test. The Kaplan–Meier method was used to estimate survival curves for all-cause mortality and respiratory failure. The log-rank test was used to compare survival curves between the progressive and non-progressive cavity groups. Univariable and multivariable logistic regression analyses were used to investigate the factors associated with cavity progression. As for the chest CT findings, presence of the largest cavity in the middle lobe or the lingula, the shortest distance from the pleura to the outer cavity wall, atelectasis, and pleural thickening were excluded from the multivariable logistic regression analysis because these were strongly correlated with consolidation around the initial cavity. Receiver Operating Characteristic (ROC) analysis was used to determine the optimal cut-off value of the maximum inner diameter of cavities for the prediction of progressive cavity (Additional file 1: Figure S1)**.** All reported *p* values were two-sided, and p values <0.05 were considered statistically significant. SAS software version 9.3 (SAS Institute, Inc., Cary, NC) was used for statistical analysis.

## Results

### Baseline patient characteristics

Of the 97 patients, 52 were in the progressive cavity group while 45 were classified to the non-progressive cavity group. Baseline patient characteristics in the two groups are summarized in Table 1. The median observation periods were 8.0 (range: 3.0–12.9) years in the progressive cavity group and 8.8 (3.8–12.0) years in the non-progressive cavity group (*p* = 0.103). The median cavity observation periods were 4.6 years (range: 1.1–11.9 years) in the progressive cavity group and 5.5 years (3.0–10.8 years) in the non-progressive cavity group (*p* = 0.025). None of the patients were known to be infected with the human immunodeficiency virus. The age at initial cavity confirmation was significantly higher in the progressive cavity group (*p* < 0.001), while BMI was significantly lower in the progressive cavity group (*p* = 0.043). In assessments of underlying lung diseases, both groups showed a high rate of bronchiectasis. However, in assessments of comorbidities, the proportion of patients with diabetes was significantly higher in the progressive cavity group (*p* = 0.005). There was no significant intergroup difference in the rates of smear positivity at initial cavity confirmation (*p* = 0.078). There was no significant intergroup difference in the rates of multidrug chemotherapy (*p* = 0.809). Glycopeptidolipid-core antibody, that is useful for diagnosing MAC-PD [15], was measured in 25 of 52 cases in the progressive group and 26 of 45 cases in the non-progressive group within 6 months of the initial CT. No significant difference in the antibody levels was observed between the two groups (5.5 U/ml [range: 2.3–11.1 U/ml] in the progressive group, 4.0 U/ml [range: 0.7–9.0 U/ml] in the non-progressive group, *p* = 0.271).

**Table 1:** Baseline characteristics of 97 patients with *Mycobacterium avium* complex pulmonary disease and cavitary lesions

Characteristic	Progressive cavity Group (*n* = 52)	Non-progressive cavity Group (*n* = 45)	*P* value
Female	42 (80.8)	38 (84.4)	0.790
Age, years	68 (54–83)	63 (51–78)	< 0.001
Body mass index, kg/m^2a^	16.7 (12.3–24.6)	18.3 (13.7–25.4)	0.043
Underlying lung disease			
Pulmonary emphysema/bulla	5 (9.6)	3 (6.7)	0.721
Old pulmonary tuberculosis	8 (15.4)	4 (8.9)	0.373
Bronchiectasis	50 (96.2)	43 (95.6)	> 0.999
Interstitial pneumonia	4 (7.7)	2 (4.4)	0.683
Comorbidity			
Chronic heart disease	15 (28.8)	14 (31.1)	0.828
Diabetes mellitus	11 (21.2)	1 (2.2)	0.005
Chronic liver disease	3 (5.8)	3 (6.7)	> 0.999
Collagen disease	3 (5.8)	2 (4.4)	> 0.999
Cerebrovascular disease	3 (5.8)	1 (2.2)	0.621
Postgastrointestinal tract surgery	3 (5.8)	3 (6.7)	> 0.999
Steroid and/or immunosuppressive agent use^b^	3 (5.9)	2 (4.4)	> 0.999
MAC species^c^			
*Mycobacterium avium*	28 (54.9)	28 (62.2)	0.536
*Mycobacterium intracellulare*	21 (41.2)	13 (28.9)	
Both	2 (3.9)	4 (8.9)	
Sputum-smear positive^d^	28 (66.7)	19 (46.3)	0.078
Treatment			
No treatment	4 (7.7)	8 (17.8)	
Non-CAM/AZM-included regimen	4 (7.7)	3 (6.7)	
Multidrug regimen^e^	41 (78.8)	34 (75.6)	0.809

Data are presented as median (range) or no. (%) of patients. *AZM* Azithromycin, *CAM* Clarithromycin, *MAC Mycobacterium avium* complex. ^a^: Body mass index was checked for 38 and 37 patients. ^b^: Steroid and/or immunosuppressive agent use was checked for 51 patients in the progressive cavity group. ^c^: MAC species was checked for 51 patients in the progressive cavity group. ^d^: Sputum-smear positivity was checked for 42 and 41 patients. ^e^: Multidrug regimen refers to CAM/AZM-containing regimen with two or more drugs for at least 1 year. *p* < 0.05 was considered significant

### Initial chest CT findings for cavities

Table 2 shows the initial chest CT findings for cavities. The distribution of cavities was significantly different between the two groups (*p* = 0.024). The maximum inner diameter of the cavity was significantly longer in the progressive cavity group (*p* < 0.001). The shortest distance from the pleura to the cavity outer wall was significantly shorter in the progressive cavity group (*p* < 0.001). The rate of multiple cavities was significantly higher in the progressive cavity group (*p* = 0.023). Table 3 shows chest CT findings observed around the initial cavity. Consolidation (*p* < 0.001), atelectasis (*p* = 0.011), and pleural thickening (*p* < 0.001) around the initial cavity were more frequently observed in the progressive cavity group.

**Table 2:** Initial chest computed tomography findings in the cavities of 97 patients

	Progressive cavity Group (*n* = 52)	Non-progressive cavity Group (*n* = 45)	*P* value
Location of the cavity			0.024
Upper lobe	30 (57.7)	20 (44.4)	
Middle lobe or lingula	2 (3.8)	10 (22.2)	
Lower lobe	20 (38.5)	15 (33.3)	
Maximum inner diameter (mm)	14.6 (4.4–55.1)	8.7 (2.6–40.4)	< 0.001
The shortest distance (mm)^a^	0.0 (0.0–24.1)	3.2 (0.0–20.9)	< 0.001
Multiple cavities	36 (69.2)	20 (44.4)	0.023

Data are presented as median (range) or no. (%) of patients. ^a^: The shortest distance is from the pleura to the outer wall of the cavity. *p* < 0.05 was considered significant

**Table 3:** Initial chest computed tomography findings around the cavities in 97 patients

	Progressive cavity Group (*n* = 52)	Non-progressive cavity Group (*n* = 45)	*P* value
Bronchiectasis	34 (65.4)	30 (66.7)	> 0.999
Small nodules	21 (40.4)	26 (57.8)	0.105
Nodules	8 (15.4)	6 (13.3)	> 0.999
Consolidation	32 (61.5)	4 (8.9)	< 0.001
Atelectasis	16 (30.8)	4 (8.9)	0.011
Pleural thickening	34 (65.4)	10 (22.2)	< 0.001
Pleural indentation	9 (17.3)	15 (33.3)	0.098
Pulmonary emphysema / bulla	2 (3.8)	1 (2.2)	> 0.999

Data are presented no. (%) of patients. *p* < 0.05 was considered significant

### Factors related to progressive cavities

**Fig. 2:**
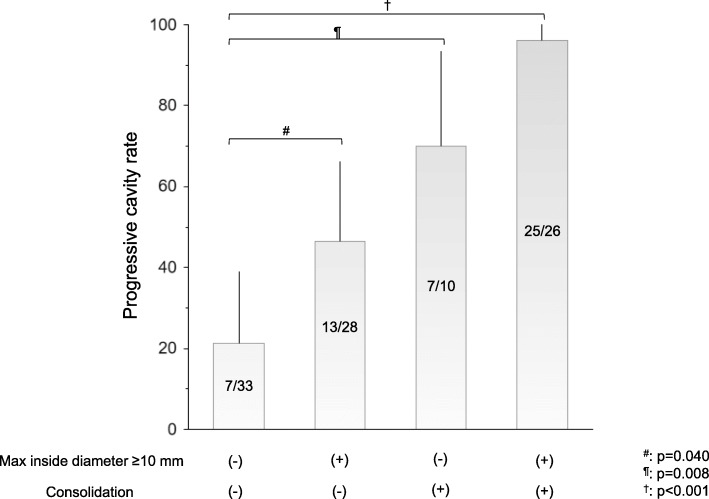
Rates of progressive cavities stratified by maximum inner diameter and consolidation around the initial cavity. Progression rates were 21.2% in patients with maximum inner diameter < 10 mm and without consolidation around the initial cavity, 46.4% in those with maximum inner diameter ≥ 10 mm and without consolidation, 70% in patients with maximum inner diameter < 10 mm and with consolidation, and 96.2% in patients with maximum inner diameter ≥ 10 mm and with consolidation. The rate of progressive cavities increased significantly with the presence of a maximum inner diameter of 10 mm and consolidation around the cavity

Table 4 shows the results of univariable and multivariable logistic regression analysis for the factors related to progressive cavities. Univariable logistic regression analysis revealed that age (*p* = 0.001), diabetes mellitus (*p* = 0.021), presence of the largest cavity in the middle lobe or lingula (*p* = 0.015), maximum internal diameter of the cavity (*p* = 0.002), the shortest distance from the pleura to the outer cavity wall (*p* = 0.010), multiple cavities (*p* = 0.015), and the findings around the cavity, namely, consolidation (*p* < 0.001), atelectasis (*p* < 0.001), and pleural thickening (*p* < 0.001), were significantly associated with progressive cavities. Multivariable logistic regression analysis revealed that consolidation around the cavity (odds ratio [OR]: 16.15, 95% confidence interval [CI]: 4.05–64.46, *p* < 0.001), age (OR: 1.12, 95% CI: 1.03–1.20, *p* = 0.005), and maximum internal diameter of the cavity (OR: 1.11, 95% CI: 1.02–1.21, *p* = 0.012) were factors that showed significant relationships with progressive cavities. Figure 2 shows the ratio of progressive cavities by the presence or absence of a cavity maximum inner diameter of ≥10 mm and consolidation around the initial cavity. Progression rates were 21.2% in patients with maximum inner diameter < 10 mm and without consolidation around the initial cavity, 46.4% in those with maximum inner diameter ≥ 10 mm without consolidation, 70% in patients with maximum inner diameter < 10 mm with consolidation, and 96.2%, in patients with maximum inner diameter ≥ 10 mm with consolidation.

**Table 4:** Factors associated with a progressive cavity

	Univariable analysis OR (95% CI)	*p* value	Multivariable analysis OR (95% CI)	*p* value
Female sex	0.77 (0.27–2.24)	0.636		
Age, years	1.11 (1.04–1.17)	0.001	1.12 (1.03–1.20)	0.005
Body mass index, kg/m^2a^	0.85 (0.71–1.01)	0.064		
Causative organism^b^				
*Mycobacterium avium*	1			
*Mycobacterium intracellulare*	1.72 (0.74–4.04)	0.211		
Sputum-smear positivity^c^	2.32 (0.95–5.63)	0.064		
Diabetes mellitus	11.80 (1.46–95.52)	0.021	7.80 (0.71–86.12)	0.094
Multidrug regimen^d^	1.21 (0.47–3.12)	0.700		
Chest CT findings at the initial cavity				
Cavity in the middle lobe or lingual	0.14 (0.03–0.68)	0.015		
Maximum inner diameter (mm)	1.12 (1.04–1.20)	0.002	1.11 (1.02–1.21)	0.012
Shortest distance (mm)^e^	0.86 (0.76–0.96)	0.010		
Multiple cavities	2.81 (1.22–6.46)	0.015	1.08 (0.35–3.34)	0.892
Chest CT findings around the initial cavity				
Consolidation	16.40 (5.10–52.78)	< 0.001	16.15 (4.05–64.46)	< 0.001
Atelectasis	4.56 (1.40–14.88)	< 0.001		
Pleural thickening	6.61 (2.67–16.35)	< 0.001		
Pleural indentation	0.42 (0.16–1.08)	0.072		

*CI* Confidence interval, *CT* Computed tomography, *OR* Odds ratio. ^a^: Body mass index was checked for 75 patients. ^b^: *Mycobacterium avium* complex species was checked for 96 patients. ^c^: Sputum-smear positivity was checked for 83 patients. ^d^: Multidrug regimen refers to CAM/AZM-containing regimen with two or more drugs for at least 1 year. ^e^: The shortest distance is from the pleura to the outer wall of the cavity. *p* < 0.05 was considered significant

### Clinical prognosis: all-cause mortality and respiratory failure

**Fig. 3:**
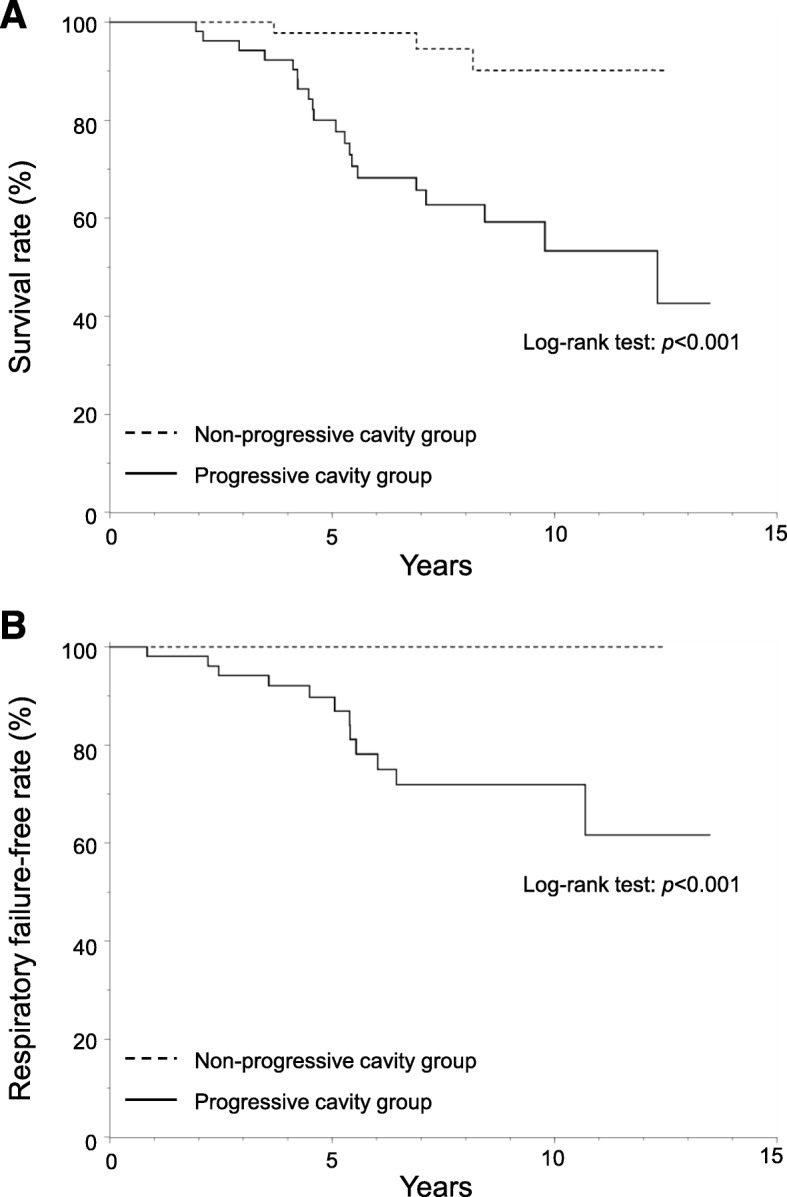
All-cause mortality and respiratory failure in progressive and non-progressive cavity groups. Panel A shows the Kaplan–Meier survival curves of all-cause mortality. Panel B shows the Kaplan–Meier survival curves of respiratory failure. *P* values from the log-rank test were used to examine differences in the Kaplan–Meier curves

Figure 3 shows the Kaplan–Meier survival curves for (A) all-cause mortality and (B) respiratory failure. The 10-year mortality rate was 46.7% in the progressive cavity group and 9.8% in the non-progressive cavity group (log-rank test: *p* < 0.001). The 10-year rate for respiratory failure was 28.1% in the progressive cavity group and 0% in the non-progressive cavity group (log-rank test: *p* < 0.001).

## Discussion

The present study revealed that large size and the presence of consolidation at an early stage were predictors of progressive cavities, which led to respiratory failure and exhibited high mortality rates in MAC-PD. In addition, when both cavity maximum inner diameter of ≥10 mm and consolidation around the cavity presented simultaneously, progressive cavities were observed with a high probability of 96.2%. A cavity is a characteristic finding that is easy to recognize on chest radiographs and CT images. The progression of the cavity leads to destruction of the lungs, which results in respiratory failure and poor prognosis. Unlike previous studies [6–9] that assessed prognostic factors but yielded recommendations that were somewhat complicated to apply in daily clinical practice, our results indicate that the combination of two simple findings can predict disease progression, which would be very useful in clinical practice. The results of this study will be of great help in decisions regarding treatment plans, including surgical intervention, when the cavity is recognized on the initial CT.

In this study, a cavity was defined as a radiographic opacity with an internal area of lucency. There are various mechanisms underlying cavitation [16]. A study showed that cavities of MAC are formed from bronchiectasis [17]. Therefore, it is difficult to exactly distinguish between cavities and bronchiectasis. In fact, the cavities found in the middle lobe or lingula in the non-progressive cavity group may be merely single enlarged bronchiectases. However, in actual clinical practice, cavities and bronchiectasis cannot be easily distinguished by CT findings. Therefore, we attempted to examine radiographic opacities with an internal area of lucency without strict distinction between cavity and bronchiectasis, except in cases of apparent bronchiectasis that exhibited beaded airspace expansion. We also did not distinguish between FC and NB disease types because progressive NB disease with cavitary lesions is essentially indistinguishable from FC disease [18].

The presence of cavitary lesion with MAC-PD reduces the effectiveness of chemotherapy. Chemotherapy for MAC-PD has improved with the introduction of macrolide antibiotics, but its effects are limited. The sputum-negative conversion rate with standard chemotherapy is 56–92%, and 11–57% of the cases show disease relapse [19–22]. The effectiveness of chemotherapy is further reduced by the presence of cavities [23, 24]. The reasons for the reduced effectiveness include the increase in bacterial growth due to the cavity aerobic environment, the reduced reach of drugs on the luminal surface, and the dissemination of bacteria to other areas in the lung from the cavity [25, 26]. A Japanese study comparing cavity sizes before and after standard chemotherapy reported 42.1% enlargement, 0.2% no-change, and 56.1% reduction, which is inferior to the treatment effect in tuberculosis more than 50 years ago [27]. Thus, patients who have MAC-PD with cavities exhibit intractable disease and require multidisciplinary treatment.

Large cavity size and consolidation around the cavity on the initial CT were predictors of disease progression. In this study, internal diameter was used to assess cavity size because the outer diameter of the cavity varies with the findings around the cavity such as consolidation, pleural thickening, and atelectasis. A smaller cavity size may represent mere bronchiectasis. Several reports have discussed the relationship between consolidation and prognosis or pathophysiology. Lee et al. [28] performed an observational study of CT findings from untreated NB MAC-PD patients with consolidation, which revealed that these patients had worse CT findings and symptoms and that they needed treatment; therefore, consolidation is an important prognostic factor. Moreover, a study [29] using anti-acid-bacterial monoclonal antibodies cross-reacting with MAC showed that the amounts of acid-fast bacteria were detected in consolidation.

The present retrospective study has several limitations. First, the timing of the initial and final CT examinations and the interval between the two examinations were not consistent because of the retrospective design. Second, in cases with multiple cavities in the initial CT, we analyzed the cavity that exhibited the largest inner diameter. Notably, even if the analyzed cavity progresses, other cavities may improve or cavities other than the analyzed cavity may progress. Thus, there may be a few cases in which analysis of the selected cavity did not necessarily reflect the patient’s prognosis. Third, chemotherapy was performed at the discretion of each attending physician for most patients, so the natural disease course could not be observed. It is possible that the cavity in the non-progressive group did not progress because the drug reached the lesion more effectively. Chemotherapy may cause changes in cavities, although the rates of chemotherapy were similar between the two groups. Finally, the definitions were arbitrarily determined because there is no standard definition of a progressive cavity at present. Nevertheless, our definition may be valid, since the progressive cavity group showed a significantly higher respiratory failure rate and worse prognosis than the non-progressive cavity group.

## Conclusions

We focused on cavities that were relatively easy to recognize on chest CT and the findings around the cavity and investigated the factors influencing progression in MAC-PD. Large cavities and cavities with consolidation were found to be predictors of high rates of mortality and respiratory failure. For cavities with these features at an early stage, multidisciplinary treatment including surgical lung resection may have to be considered. Further prospective studies are needed to confirm these conclusions.

## Supplementary information

**Additional file 1: Figure S1.** Receiver Operating Characteristic (ROC) curve of the maximum inner diameter of cavities in the prediction of progressive cavity. The area under the ROC curve was 0.74 (95% CI: 0.64–0.84). The optimal threshold for the prediction of progressive cavity was 10 mm, with 73.1% sensitivity and 64.4% specificity.

## Data Availability

The data are not available for public access because of patient privacy concerns but are available from the corresponding author on reasonable request.

## Abbreviations

AZM: Azithromycin
BMI: Body mass index
CAM: Clarithromycin
CI: Confidence interval
CT: Computed tomography
FC: Fibrocavitary
HU: Hounsfield unit
MAC-PD: *Mycobacterium avium* complex pulmonary disease
NB: Nodular/bronchiectatic
NTM-PD: Nontuberculous mycobacteria pulmonary disease
OR: Odds ratio

## References

[1] Adjemian J, Olivier KN, Seitz AE, Holland SM, Prevots DR (2012). Prevalence of nontuberculous mycobacterial lung disease in U.S. Medicare beneficiaries. Am J Respir Crit Care Med.

[2] Khan K, Wang J, Marras TK (2007). Nontuberculous mycobacterial sensitization in the United States: national trends over three decades. Am J Respir Crit Care Med.

[3] Thomson RM (2010). NTM working group at Queensland TB control Centre and Queensland mycobacterial reference laboratory. Changing epidemiology of pulmonary nontuberculous mycobacteria infections. Emerg Infect Dis.

[4] Hoefsloot W, van Ingen J, Andrejak C, Angeby K, Bauriaud R, Bemer P (2013). Nontuberculous mycobacteria network European trials group. The geographic diversity of nontuberculous mycobacteria isolated from pulmonary samples: an NTM-NET collaborative study. Eur Respir J.

[5] Namkoong H, Kurashima A, Morimoto K, Hoshino Y, Hasegawa N, Ato M (2016). Epidemiology of pulmonary nontuberculous mycobacterial disease. Japan Emerg Infect Dis.

[6] Hayashi M, Takayanagi N, Kanauchi T, Miyahara Y, Yanagisawa T, Sugita Y (2012). Prognostic factors of 634 HIV-negative patients with Mycobacterium avium complex lung disease. Am J Respir Crit Care Med.

[7] Kumagai S, Ito A, Hashimoto T, Marumo S, Tokumasu H, Kotani A (2017). Development and validation of a prognostic scoring model for Mycobacterium avium complex lung disease: an observational cohort study. BMC Infect Dis.

[8] Gochi M, Takayanagi N, Kanauchi T, Ishiguro T, Yanagisawa T, Sugita Y (2015). Retrospective study of the predictors of mortality and radiographic deterioration in 782 patients with nodular/bronchiectatic Mycobacterium avium complex lung disease. BMJ Open.

[9] Hwang JA, Kim S, Jo KW, Shim TS (2017). Natural history of Mycobacterium avium complex lung disease in untreated patients with stable course. Eur Respir J.

[10] Ahn CH, McLarty JW, Ahn SS, Ahn SI, Hurst GA (1982). Diagnostic criteria for pulmonary disease caused by Mycobacterium kansasii and Mycobacterium intracellulare. Am Rev Respir Dis.

[11] Research Committee of the British Thoracic Society (2002). Pulmonary disease caused by Mycobacterium avium-intracellulare in HIV-negative patients: five-year follow-up patients receiving standardized treatment. Int J Tuberc Lung Dis.

[12] Haworth CS, Banks J, Capstick T, Fisher AJ, Gorsuch T, Laurenson IF (2017). British Thoracic Society guidelines for the management of non-tuberculous mycobacterial pulmonary disease (NTM-PD). Thorax.

[13] Asakura T, Hayakawa N, Hasegawa N, Namkoong H, Takeuchi K, Suzuki S (2017). Long-term outcome of pulmonary resection for nontuberculous mycobacterial pulmonary disease. Clin Infect Dis.

[14] Griffith DE, Aksamit T, Brown-Elliott BA, Catanzaro A, Daley C, Gordin F (2007). An official ATS/IDSA statement: diagnosis, treatment, and prevention of nontuberculous mycobacterial diseases. Am J Respir Crit Care Med.

[15] Kitada S, Kobayashi K, Ichiyama S, Takakura S, Sakatani M, Suzuki K (2008). Serodiagnosis of Mycobacterium avium-complex pulmonary disease using an enzyme immunoassay kit. Am J Respir Crit Care Med.

[16] Gadkowski LB, Stout JE (2008). Cavitary pulmonary disease. Clin Microbiol Rev.

[17] Kim TS, Koh WJ, Han J, Chung MJ, Lee JH, Lee KS (2005). Hypothesis on the evolution of cavitary lesions in nontuberculous mycobacterial pulmonary infection: thin-section CT and histopathologic correlation. AJR Am J Roentgenol.

[18] Aksamit TR (2002). Mycobacterium avium complex pulmonary disease in patients with pre-existing lung disease. Clin Chest Med.

[19] Dautzenberg B, Piperno D, Diot P, Truffot-Pernot C, Chauvin JP (1995). Clarithromycin study Group of France. Clarithromycin in the treatment of Mycobacterium avium lung infections in patients without AIDS. Chest.

[20] Wallace RJ, Brown BA, Griffith DE, Girard WM, Murphy DT (1996). Clarithromycin regimens for pulmonary Mycobacterium avium complex. The first 50 patients. Am J Respir Crit Care Med.

[21] Tanaka E, Kimoto T, Tsuyuguchi K, Watanabe I, Matsumoto H, Niimi A (1999). Effect of clarithromycin regimen for Mycobacterium avium complex pulmonary disease. Am J Respir Crit Care Med.

[22] Kobashi Y, Matsushima T (2003). The effect of combined therapy according to the guidelines for the treatment of Mycobacterium avium complex pulmonary disease. Intern Med.

[23] Fujiuchi S, Matsumoto H, Yamazaki Y, Nakao S, Takahashi M, Satoh K (2003). Analysis of chest CT in patients with Mycobacterium avium complex pulmonary disease. Respiration.

[24] Kuroishi S, Nakamura Y, Hayakawa H, Shirai M, Nakano Y, Yasuda K (2008). Mycobacterium avium complex disease: prognostic implication of high-resolution computed tomography findings. Eur Respir J.

[25] Canetti G (1965). Present aspects of bacterial resistance in tuberculosis. Am Rev Respir Dis.

[26] Palaci M, Dietze R, Hadad DJ, Ribeiro FK, Peres RL, Vinhas SA (2007). Cavitary disease and quantitative sputum bacillary load in cases of pulmonary tuberculosis. J Clin Microbiol.

[27] Kurashima A, Horibe M (2012). Distribution of pulmonary Mycobacterium avium complex (MAC) disease cavities and their course under chemotherapy. Kekkaku.

[28] Lee G, Lee KS, Moon JW, Koh WJ, Jeong BH, Jeong YJ (2013). Nodular bronchiectatic Mycobacterium avium complex pulmonary disease. Natural course on serial computed tomographic scans. Ann Am Thorac Soc.

[29] Hibiya K, Shigeto E, Iida K, Kaibai M, Higa F, Tateyama M (2012). Distribution of mycobacterial antigen based on differences of histological characteristics in pulmonary Mycobacterium avium infectious diseases--consideration of the extent of surgical resection from the pathological standpoint. Pathol Res Pract.

