# Supplementary material for: Characteristic chest CT findings for progressive cavities in Mycobacterium avium complex pulmonary disease: a retrospective cohort study
Source: Respir Res. 2020 Jan 8;21:10. doi: 10.1186/s12931-020-1273-x (PMC6950929; doi:10.1186/s12931-020-1273-x)
Supplement: Supplementary file 1 [file 12931_2020_1273_MOESM1_ESM.docx]

Figure 1S

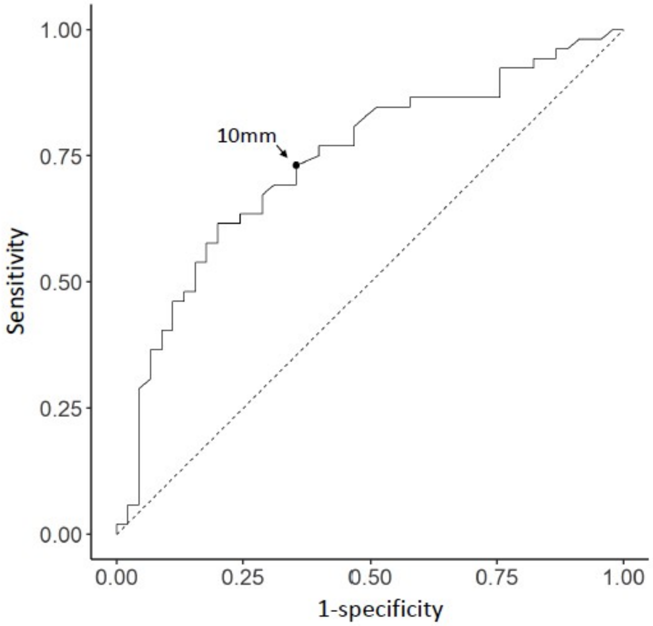

1.00
0.75
0.50
0.25
0.00
Sensitivity
10mm
0.00
0.25
0.50
0.75
1.00
1-specificity
